# Client uptake of safer conception strategies: implementation outcomes from the Sakh’umndeni Safer Conception Clinic in South Africa

**DOI:** 10.7448/IAS.20.2.21291

**Published:** 2017-03-08

**Authors:** Sheree R Schwartz, Jean Bassett, Charles B Holmes, Nompumelelo Yende, Rebecca Phofa, Ian Sanne, Annelies Van Rie

**Affiliations:** ^a^ Department of Epidemiology, Johns Hopkins School of Public Health, Baltimore, MD, USA; ^b^ Witkoppen Health and Welfare Centre, Johannesburg, South Africa; ^c^ Centre for Infectious Disease Research in Zambia, Lusaka, Zambia; ^d^ Johns Hopkins School of Medicine, Lusaka, Zambia; ^e^ Clinical HIV Research Unit, Department of Medicine, University of the Witwatersrand, Johannesburg, South Africa; ^f^ Right to Care, Johannesburg, South Africa; ^g^ Department of Epidemiology, University of North Carolina at Chapel Hill, Chapel Hill, NC, USA; ^h^ Department of Epidemiology and Social Medicine, University of Antwerp, Antwerpen, Belgium

**Keywords:** HIV prevention, safer conception, serodiscordance, pre-exposure prophylaxis, self-insemination, timed condomless sex, implementation, sub-Saharan Africa

## Abstract

**Introduction:** Implementation of safer conception services for HIV-affected couples within primary healthcare clinics in resource-limited settings remains limited. We review service utilization and safer conception strategy uptake during the first three years of *Sakh’umndeni*, which is a safer conception clinic in South Africa.

**Methods:** *Sakh’umndeni* is located at Witkoppen Health and Welfare Centre, a high-volume primary healthcare clinic in northern Johannesburg. Men and women desiring to conceive in less than or equal to six months and in relationships in which one or both partners are living with HIV are eligible for safer conception services. Clients receive a baseline health assessment and counselling around periconception HIV risk reduction strategies and choose which strategies they plan to use. Clients are followed-up monthly. We describe client service utilization and uptake and continuation of safer conception methods. Factors associated with male partner attendance are assessed using robust Poisson regression.

**Results:** Overall 440 individuals utilized the service including 157 couples in which both partners attended (55%) and 126 unaccompanied female partners. Over half of the couples (55%) represented were in serodiscordant/unknown status relationships. Higher economic status and HIV-negative status of the women increased male partner involvement, while HIV-negative status of the men decreased male involvement. Regarding safer conception strategies, uptake of antiretroviral therapy initiation (90%), vaginal self-insemination among partnerships with HIV-negative men (75%) and timed condomless intercourse strategies (48%) were variable, but generally high. Overall uptake of pre-exposure prophylaxis (PrEP) was 23% and was lower among HIV-negative men than women (7% vs. 44%, *p* < 0.001). Male medical circumcision (MMC) was used by 28% of HIV-negative men. Over 80% of clients took up at least one recommended safer conception strategy. Continuation of selected strategies over attempted conception attempts was >60%.

**Conclusions:** Safer conception strategies are generally used by clients per recommendations. High uptake of strategies suggests that the proposed combination prevention methods are acceptable to clients and appropriate for scale-up; however, low uptake of PrEP and MMC among HIV-negative men needs improvement. Targeted community-based efforts to reach men unlinked to safer conception services are needed, alongside streamlined approaches for service scale-up within existing HIV and non-HIV service delivery platforms.

## Introduction

Efforts to conceptualize frameworks for safer conception care have increased in recent years in response to the realization that many individuals and couples living with or affected by HIV hope to have a child in the future [1–6]. Safer conception strategies for resource-limited settings have been well documented and include antiretroviral therapy (ART) for HIV-positive partners, pre-exposure prophylaxis (PrEP) for HIV-negative partners, male medical circumcision (MMC) for HIV-negative men and reduced exposure to HIV during attempted conception through vaginal self-insemination (if the male partner is HIV-negative) or timed condomless intercourse limited to windows of peak female fertility [3,7–9].

Formative work from across sub-Saharan Africa suggests that healthcare providers do not have the knowledge necessary to provide safer conception counselling and care, resulting in low self-efficacy around administration of safer conception counselling [10–13]. Consequently, provider efforts tend to be limited to maternal health and prevention of vertical transmission of HIV [10,12,14]. As a result, patients have limited knowledge of safer conception methods, which limits their uptake of safer conception strategies and their ability to reduce HIV transmission risks [15–17]. Qualitative studies have indicated that the proposed safer conception strategies are acceptable to clients [18,19]. However, quantitative data documenting uptake of strategies among safer conception clients in resource-limited settings remain scarce [20].

We report implementation data from the first publicly available safer conception demonstration project set in a primary healthcare setting in South Africa. The objective of this analysis is to review implementation outcomes from the first three years of service delivery. We assess service utilization, including who accesses the services and who is missing from care, and uptake of safer conception strategies by type of client. Lessons learned from this specialized service delivery model are critical to inform future scale-up of safer conception care within the region.

## Methods

### Study design and setting

*Sakh’umndeni* is a prospective demonstration project offering a one-stop shop for safer conception services. HIV-affected individuals of reproductive age (women 18–49 years and men 18–59 years) in relationships in which one or both partners are HIV-positive and who want to have a child within the next six months are eligible for care at the *Sakh’umndeni* Safer Conception Clinic. The nurse-run service is located at Witkoppen Health and Welfare Centre (Witkoppen Clinic), a high-volume, primary health centre located in northern Johannesburg and serving the surrounding informal settlements. Witkoppen Clinic offers comprehensive outpatient services, including HIV counselling and testing (HCT), ART initiation and management, chronic and acute adult and paediatric care and antenatal/postnatal care. Witkoppen Clinic charges clients ZAR 55 (approximately USD 3.50), though the fee is waived if clients are unable to pay. Safer conception services were introduced at Witkoppen Clinic in July 2013 and continue to operate through funding from the United States Agency for International Development (USAID) and the President’s Emergency Plan For AIDS Relief (PEPFAR), in partnership with the South African Department of Health.

The *Sakh’umndeni* approach to safer conception care has been described previously [21]. Briefly, each individual or couple seeking services undergoes a medical examination and reproductive health history, including HCT for the HIV-negative partner, syndromic screening for sexually transmitted infections (STIs) and syphilis testing and HIV viral load monitoring for HIV-positive partners. Following the baseline assessment, clients receive counselling around available periconception HIV prevention methods, including the risks and benefits of each method given the couples’ HIV status. Methods counselled include HCT for HIV-negative partners and those with unknown status, ART initiation for HIV-positive partners independent of immunologic or clinical staging, PrEP for HIV-negative partners, self-insemination using a syringe or turkey baster (specifically encouraged when the male partner is HIV-negative), MMC and timed condomless intercourse limited to the monthly time frame of peak fertility. Clients are informed of all the methods and given tailored counselling and recommended strategies based on their individual and relationship dynamics. Clients then choose which strategies they plan to use and receive ongoing counselling during follow-up; clients may elect to change strategies used over time. All services are provided at *Sakh’umndeni*, with the exception of MMC which is provided on site at Witkoppen Clinic for free. Clients are prospectively followed up monthly during which repeat HCT for HIV-negative partners, pregnancy testing, safer conception counselling and STI screening are conducted, alongside viral load monitoring for individuals who are not suppressed two months following ART initiation or step-up adherence counselling. Once a couple is virally suppressed and comfortable/adherent to their selected strategies, they are determined to be ready for conception, and they start a six-month trial period. When only one partner attends the clinic, we work with the attending partner to bring the absent partner into care, and when attendance cannot be facilitated, we work to motivate for HIV testing and care for the absent partner prior to encouraging attempted conception. For couples in which this cannot be navigated and pregnancy is still going to be attempted, we offer PrEP if the attending partner is HIV-negative. Those not conceiving during the trial period, continue their ART care at Witkoppen Clinic and are referred to fertility services if infertility is suspected. We elected to include women up to age 49 years as they fall within the age range of women of reproductive age. However, as conception probabilities decrease with age, our providers screen for signs of menopause or infertility prior to enrolling for services and counsel older women who are still regularly menstruating of their reduced conception chances in order to help them weigh the risks and benefits associated with attempted conception.

All participants provided informed consent. This study was approved by the Human Research Ethics Committee at the University of the Witwatersrand in Johannesburg, South Africa, the Institutional Review Boards at the University of North Carolina and the Johns Hopkins School of Public Health.

### Data collection and analysis

Demographics and relevant social and clinical histories, as well as laboratory results, baseline and follow-up safer conception counselling and utilization of strategies are collected using standard case report forms. Individuals unaccompanied by their partner answer relevant questions about their partner’s demographics, health and reproductive history. Data were entered into a REDCap database (Vanderbilt, TN, USA) hosted by Witkoppen Health and Welfare Centre and analysed using Stata 14.1 (College Station, TX, USA).

As this is an ongoing implementation study, follow-up times for participants vary and may exceed six months, given that attempted conception is only recommended after the clients are clinically indicated for conception. We present data for clients attending the service from July 2013 through a cutoff date of May 2016. The overall study will be completed in the following year.

We present descriptive statistics of the study population, measures of uptake of safer conception strategies, as well as continuation of strategies over time. Chi-squared (*Χ*^2^) or Fischer exact statistics were used for comparisons between groups. Prevalence ratios (PRs) of factors associated with male partner attendance at *Sakh’umndeni* were assessed among female clients using robust Poisson regression, as the prevalence of male partner attendance exceeded 10% [22]. Men who attended the clinic at any time were considered to have attended. In a clinical trial setting, it would be expected that men attend each visit. In the real world, however, we encourage partners to attend each visit together, but we recognize that once the male partner’s HIV status is confirmed, he is virally suppressed (if HIV-positive) and counselling around methods has been received, that the woman may attend several visits alone and the male partner will return primarily for ART management or HIV re-testing and potentially PrEP if HIV-negative. Getting men into the clinic initially is the most important and challenging step; thus, we have focused the analyses on whether the male partner ever attended the clinic. Variables that we posited may be associated with male partner attendance at the individual level included women’s age, employment status, monthly income, education, nationality (South African vs. non-native South African), number of children and HIV status. Partnership characteristics considered included whether the couple had a child together, whether the man had any living children from any woman, difference in age between partners, relationship duration and the male partners’ HIV status. Factors associated with male partner attendance at the *p* < 0.10 level in univariate analysis were included in the multivariate analysis unless strong correlations existed between the individual- and partner-level data. We conducted a complete case analysis as missing data were less than 5%.

## Results

Between July 2013 and May 2016, 440 adults enrolled into care at *Sakh’umndeni*. The majority of clients (75%) were referred to *Sakh’umndeni* from other service providers at Witkoppen Clinic or saw fliers posted at the clinic, while the remaining 25% were referred from other clinics in Johannesburg or through media campaigns. The service was run by a senior nurse and counsellor. The nurse consulted with a doctor about clients with prevalent hepatitis B infection; clients with fibroids, genital warts or polyps were referred to tertiary care for treatment prior to attempted conception.

### Service utilization

**Figure 1.**
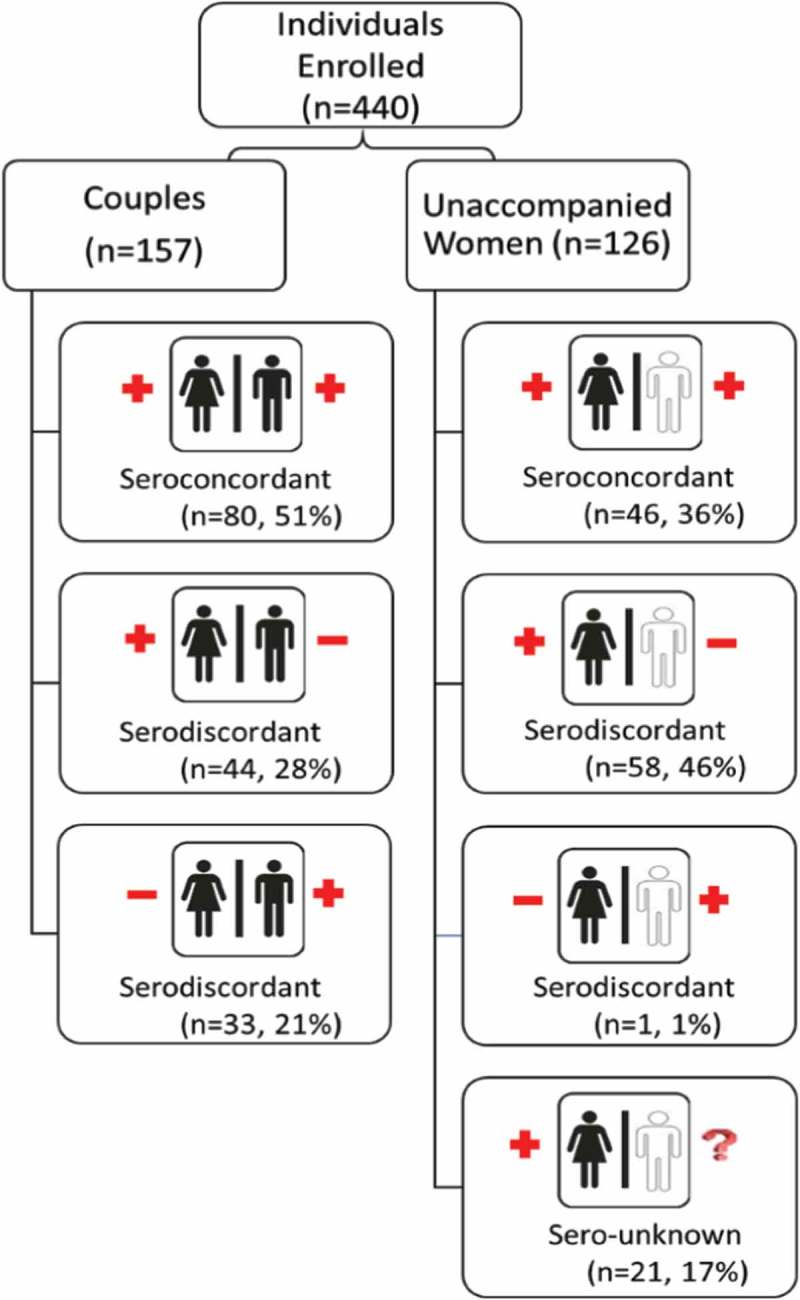
Utilization of safer conception services by couples’ HIV status. HIV status confirmed for both partners among enrolled couples; partner HIV status as reported by female partner among unaccompanied women enrolled in the service.

The 440 individual clients attending *Sakh’umndeni* represent 283 couples: 157 couples in which both partners ever attended and 126 couples for whom only the woman attended and were always unaccompanied by her male partner. Overall, 48% of the 283 couples were confirmed through HIV testing to be in serodiscordant relationships or believed their partner to be HIV-negative (in the case of unaccompanied women), whereas 45% were in seroconcordant relationships and 7% of couples were unsure of one partners’ serostatus. The HIV dynamics of the relationships, including which partner in serodiscordant relationships was living with HIV, are presented in Figure 1. The distribution of serostatus dynamics differed between women who did and did not attend with their male partners (Figure 1, *p* < 0.001). Women attending with their male partners were more commonly in seroconcordant relationships (51% vs. 36%), and 17% of female clients unaccompanied by their male partner did not know the status of their male partner. The inclusion of serodiscordant partners was comparable across the two groups (49% in which the male partner attended and 47% in which the male partner did not attend).

Characteristics of individuals utilizing the service are presented in Table 1. The majority of clients were employed and were South African, though over one-third (37%) of clients were from other African countries. Fifty-nine per cent of women and 70% of men had at least one living child; 72% of couples with children had at least one child together. The average relationship duration was 5 years (interquartile range (IQR): 2–10). At the time of enrolment into the safer conception service, 85% of women living with HIV and 73% of men living with HIV were on ART; however, 33% of HIV-positive women and 53% of HIV-positive men had detectable viral loads (≥50 copies/ml). The mean and median viral loads among unsuppressed women were 3532 (standard deviation (sd) 15,960) and 201 copies/ml (IQR: 83–545), respectively; similarly among men, the mean and median viral loads were 6766 (sd 19,026) and 196 copies/ml (IQR: 68–585), respectively. Prevalence of symptomatic STIs at enrolment was low, 4% among women and 3% among men.

**Table 1.** Characteristics of Sakh’umndeni Safer conception service clients at Witkoppen Health and Welfare Centre, Johannesburg, South Africa, 2013–2016 (*n* = 440)

Client characteristics	Women (*n* = 283)	Men (*n* = 157)
*Demographics*		
Age, median (IQR)	34 (30–38)	38 (34–43)
Employed, *n* (%)	182 (64)	131 (83)
Average monthly income, median USD (IQR)	267 (167–400)	273 (167–467)
Education, *n* (%)		
Primary or below	60 (21)	39 (25)
Some high school	128 (45)	50 (32)
Matriculated high school	95 (34)	68 (43)
Nationality, *n* (%)		
South African	177 (63)	99 (63)
Non-native South African	106 (37)	42 (27)
*Reproductive history*		
Ever been pregnant, *n* (%)	217 (77)	–
Number of living children, median (IQR)	1 (0–1)	1 (0–2)
Number of living children, *n* (%)		
None	115 (41)	47 (30)
One	117 (41)	47 (30)
Two or more	51 (18)	63 (40)
*Clinical characteristics*		
BMI, median (IQR)	26.5 (23.5–31.7)	25.5 (23.0–28.7)
STI at enrolment, *n* (%)	11 (3.9)	5 (3.2)
Circumcised, *n* (%)		
Not circumcised	–	81 (52)
Medically circumcised	–	26 (16)
Traditionally circumcised	–	50 (32)
HIV status, *n* (%)		
HIV-positive	249 (88)	113 (72)
HIV-negative	34 (12)	44 (28)
On antiretroviral therapy^a^, *n* (%)	211 (85)	82 (73)
Baseline viral load undetectable^b^, *n* (%)	141 (67)	29 (47)
HIV status within the partnership, *n* (%)		
HIV seroconcordant	126 (45)	80 (51)
HIV serodiscordant	136 (48)	77 (49)
HIV status of one partner unknown	21 (7)	0 (0)

IQR: interquartile range; BMI: body mass index; STI: sexually transmitted infection. ^a^Among HIV-positive clients. ^b^Baseline viral loads available for *n* = 210 women and *n* = 62 men.

Only 55% (*n* = 157/283) of women were ever accompanied by their male partners, while 45% always attended alone. We thus looked at correlates of male partner attendance to understand who is not utilizing the service (Table 2). In the univariate analyses, women who were 35 years or older, unemployed, of a higher income or HIV-negative were more likely to attend the clinic with their male partner. Conversely, women with male partners who were HIV-negative or of unknown status or male partners who have living children were less likely to be accompanied by their male partner. In multivariate analysis, female unemployment and higher income remained associated with male partner attendance. HIV-negative women had a 44% higher prevalence of male partner attendance than HIV-positive women (PR: 1.44, 95% confidence interval (CI): 1.23–1.68, *p* < 0.001) and correspondingly women with male partners who were HIV-negative or of an unknown status were 41% less likely to attend the clinic with a male partner (PR: 0.59, 95% CI: 0.46–0.77, *p* < 0.001). Women whose partners already had a living child remained less likely to attend the safer conception clinic with their male partner. In a separate exploratory analysis assessing the importance of serodynamics rather than individual HIV status, serodiscordance was not associated with male partner attendance (*results not shown)*.

**Table 2.** Correlates of male partner engagement in safer conception services among women enrolled in care at Sakh’umndeni (*n* = 283)

Characteristics	Prevalence ratio (95% CI)	*p*-Value	Adjusted prevalence ratio (95% CI)	*p*-Value
*Female partner characteristics*				
Age				
<35 years	REF		REF	
≥35 years	1.25 (1.00–1.55)	0.047	1.03 (0.84–1.27)	0.757
Employment				
Employed	0.73 (0.59–0.89)	0.002	0.75 (0.62–0.91)	0.003
Unemployed	REF		REF	
Income^a^				
Monthly income < USD 350	REF		REF	
Monthly income ≥ USD 350	1.25 (1.02–1.55)	0.036	1.26 (1.04–1.54)	0.021
Education				
Primary or below	REF			
Some high school	0.86 (0.66–1.12)	0.265		
Matriculated high school	0.96 (0.74–1.26)	0.795		
Nationality				
South African	0.99 (0.80–1.23)	0.962		
Non-native South African	REF			
Has any living children				
Yes	1.10 (0.89–1.38)	0.362		
No	REF			
HIV status				
HIV-positive	REF		REF	
HIV-negative	1.95 (1.70–2.24)	<0.001	1.44 (1.23–1.68)	<0.001
*Couple and male partner characteristics*^b^				
Couple has a child together^a^				
Yes	1.00 (0.79–1.27)	0.985		
No	REF			
Male partner >5 years older than woman^a^				
Yes	1.17 (0.95–1.44)	0.131		
No	REF			
Relationship duration, years^a^	1.01 (0.99–1.03)	0.312		
Male partner has any living children^a^				
Yes	0.73 (0.60–0.89)	0.002	0.72 (0.60–0.86)	<0.001
No	REF		REF	
Male HIV status				
HIV-positive	REF		REF	
HIV-negative/unknown	0.51 (0.39–0.66)	<0.001	0.59 (0.46–0.77)	<0.001

CI: confidence interval. ^a^Missing data: income (*n* = 3), couple has a child together (*n* = 5), male partner age (*n* = 3), relationship duration (*n* = 5) and male partner has any living children (*n* = 9). Final multivariate model, *n* = 271. ^b^Women whose partners did not attend the clinic reported characteristics of their male partners.

We also explored average time-to-male partner participation among all men attending the clinic and reasons provided by women for their partners’ absence. On average, men enrolled in the safer conception clinic on the same day as their female partners (median 0 days difference (IQR 0–0)). Men who initially did not accompany their partner, enrolled a median of 45 days later (IQR 23–140). “Having to work or no time” was cited by 63% of women as the primary reason that their partner had not attended the clinic (*n* = 79/126). Other common reasons were that she attended the general clinic alone and was referred to the safer conception clinic (22%, *n* = 28/126) and that the male partner lives in another province/country (11%, *n* = 14/126). No women indicated that lack of disclosure of HIV status to their partner or lack of desire for a child by the partner as reasons for his absence. Among women living with HIV, reported disclosure of HIV status to male partners was high among both women with partners attending and not attending the clinic (91% vs. 96%, respectively, *p* = 0.294).

### Uptake and continuation of safer conception strategies

The average number of visits per couple (a female visit, male visit or combined visit) was 12 (IQR: 8–12). Men attending the service had a median of 3 visits (IQR: 1–5). Most (83%, *n* = 235/283) couples represented had at least one follow-up visit. HCT was provided to all HIV-negative clients. Eleven participants tested HIV-positive at enrolment, including 1 out of 35 women (3%) and 10 out of 54 men (19%) without a prior HIV diagnosis.

**Figure 2.**
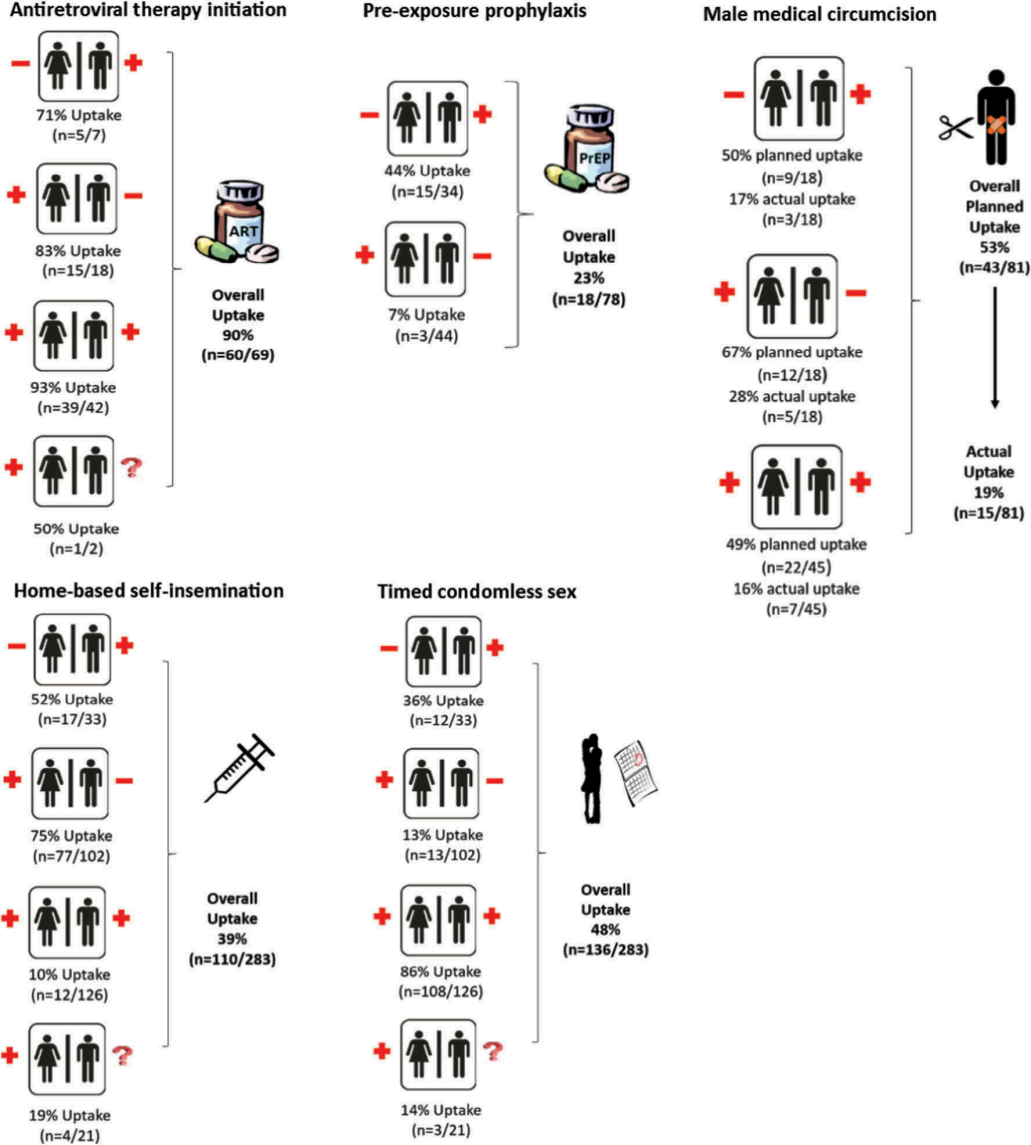
Uptake of safer conception HIV prevention strategies among Sakh’umndeni clients. Overall uptake of five safer conception strategies among eligible clients is presented, as well as uptake according to the HIV dynamics of the relationship. Clients can utilize more than one safer conception strategy. Women enrolled as couples and women unaccompanied by their male partners are presented together in this figure.

Uptake of safer conception strategies overall and by HIV serostatus is presented in Figure 2. Ninety per cent (*n* = 60/69) of ART-naive individuals initiated ART as part of the safer conception services. Uptake of ART among HIV-positive clients was similar among women and men (33/38 or 87% vs. 27/31 or 87%, *p* = 1.0). PrEP uptake among HIV-negative clients was low at 23% (*n* = 18/78), but higher among women than men (44% vs. 7%, *p*<0.001). All women choosing to initiate PrEP were attending the clinic with their male partners. Among women initiating PrEP, 67% (*n* = 10/15) of the women’s HIV-positive partners were on ART, but just 50% were virally suppressed at enrolment; similarly among men initiating PrEP, 67% (*n* = 2/3) of female partners were on ART and 33% were virally suppressed. At enrolment, 76/157 (48%) men attending the safer conception clinic were already circumcised. Among those not circumcised, 53% expressed interest and planned to undergo MMC; however, actual uptake was just 19%. MMC uptake was higher, but not statistically significantly greater, among HIV-negative compared to HIV-positive men (28% vs. 16%, *p* = 0.305). Home-based self-insemination using a syringe or turkey baster was chosen as a safer conception strategy by 39% of couples. Uptake was appropriately highest (75%) among couples with HIV-negative male partners. Even though this method is not the preferred method for discordant couples in which the male partner was HIV-positive, 52% of these couples chose to use this method. Timed condomless sex during the monthly time frame of peak fertility was used by 48% of clients, though uptake varied substantially according to the HIV dynamics within the relationship. Uptake was very high among HIV-seroconcordant couples (86%), but low (13%) in serodiscordant partnerships with an HIV-negative male partner.

Overall 80% of couples (*n* = 227/283) chose at least one recommended safer conception strategy, 5% (*n* = 14) remained undecided and 15% (*n* = 42) had not taken up any of the optimal recommended safer conception strategies. Among those 42 not starting at least one new recommended safer conception strategy, 39/42 (93%) couples had at least one HIV-positive partner on ART and 50% had a known, suppressed viral load (21/42) at enrolment. Twenty-four per cent (*n* = 68/283) chose to use two or more new safer conception strategies. In terms of combinations, 12% employed ART initiation and timed condomless sex, while 8% relied on ART initiation and self-insemination; other combinations like PrEP use and self-insemination or ART initiation of one partner and PrEP use of the other partner were less common (2% each). Twelve per cent (*n* = 34/283) elected to use a suboptimal strategy (MMC or self-insemination when the male partner was HIV-positive). Of those, 8/34 (24%) also chose one recommended strategy as well.

In terms of safer conception strategy continuation, 82% (*n* = 49/60) of clients initiated on ART were retained in care. PrEP was continued throughout the study for all clients electing this method, as measured by prescriptions from the safer conception clinic and pharmacy refills. For participants with more than six months of follow-up, self-insemination was continued for 69.1% of follow-up visits, and continuation of timed condomless sex across follow-up visits was 75.4%.

## Discussion

These are the first comprehensive implementation data with longitudinal follow-up to emerge from a safer conception service in sub-Saharan Africa, offering clear lessons for future service delivery.

Over half of individuals using the service were in serodiscordant relationships or relationships in which the status of one partner is unknown. This number has grown from 39% in the first year of implementation to 61% in the last year, suggesting that over time the service has more effectively reached those with greater safer conception needs [21]. Men remain challenging to engage in care. Our findings indicate that the men most likely to fail to attend are those in which the female partner is HIV-positive, as well as relationships in which the male partners’ status is unknown or presumed to be HIV-negative. We work closely with individuals to help facilitate attendance of both partners, including phone calls to the male partner and recommendations of alternative locations for HIV testing. We also work with women to disclose their status to the male partner if this has not previously occurred. We encourage but do not force HIV disclosure. Some women have brought their male partners and asked our service providers to assist with HIV disclosure, while in other cases we have provided couples counselling and testing. Our HIV counsellor has received in-service training on disclosure, including role-playing techniques, and works with clients over time on this process; furthermore, our service benefits from an HIV social worker and psychologist on site who work with couples post-disclosure when further counselling is required. Given that the female partner is more likely to be HIV-positive when the male partner is absent, her attendance is still beneficial for both partners as it can lead to ART initiation, alongside viral load monitoring and/or instruction around self-insemination or timed condomless sex, which together can work to prevent horizontal transmission to the male partner despite his absent attendance. At enrolment, many individuals were not virally suppressed, and our service provides an opportunity for adherence counselling, as well as counselling to delay conception until viral suppression has been achieved. These benefits notwithstanding, men with HIV-negative or unknown status are those men most likely to benefit from safer conception services and may have or acquire undiagnosed infection requiring care to optimize the health of the family prior to conception. Novel and creative efforts to reach these men must be developed. Targeted community-based HCT and home-based safer conception counselling for these men at high risk of HIV infection may be appropriate to help reach these partners and link them to services.

High uptake and continuation of safer conception strategies supports the notion that the proposed combination prevention package is acceptable and has durability in its impact among clients over time. Our findings of actual strategies selected by participating women and couples confirm the formative work conducted prior to study launch, which suggested that self-insemination, rather than PrEP, was the preferred method for HIV-negative men [18]. Our results also reinforce qualitative findings from another South African study, which reported clients’ distrust of condomless sex for fear of HIV transmission, unless they are in a concordant partnership [23]. Although PrEP uptake overall was relatively low (23%), this finding is not worrisome, given recent modelling studies which suggest minimal additional benefit of PrEP when the HIV-positive partner is on ART [24]. PrEP uptake among women was higher than expected (44%), and further qualitative work is warranted to understand what is driving women’s decisions to use PrEP when their male partner is also enrolled in the service. Whether clients are using PrEP as additional protection due to risk aversion or as a bridging strategy due to the lack of trust in their partner to adhere to treatment requires further investigation.

Although in general uptake of safer conception strategies has been appropriate per recommendations and much higher than that observed elsewhere in the absence of safer conception services [16,25], a clearer understanding of why couples at times choose less optimal strategies for their situation is warranted. In particular, it is unclear why couples in serodiscordant relationships in which the male partner is HIV-positive are choosing to use self-insemination, a less efficient conception method which is not recommended as it has no HIV transmission benefits in this situation. Clarity over clients’ understanding of the benefits is necessary to determine if they misinterpreted counselling or ultimately are acting out of the belief that anything is better than condomless sex.

Despite the uniqueness of this study, there are also limitations. This study was conducted in a real-world setting but is a specialized service with dedicated staff. As future scale-up models are unlikely to have staff dedicated to safer conception provision, counselling and fidelity of intervention implementation may be less consistent, and uptake of safer conception methods may be lower than what was observed in this setting. Furthermore, we rely on self-report of method uptake and use, and objective adherence measures to safer conception strategies were not assessed. While HIV prevention and pregnancy-related outcomes from this demonstration project have yet to be reported and thus effectiveness was not assessed in this analysis, many lessons can be learned through the assessment of implementation indicators, including service utilization and the uptake and continuation of safer conception strategies.

## Conclusions

There is a critical need for next-generation HIV combination prevention methods, such as safer conception approaches, to sustainably reduce new infections while meeting people’s reproductive desires. Our experience with *Sakh’umndeni* suggests that the methods promoted are appropriate for clients in a sub-Saharan African setting and will be used when healthcare workers offer them. Indeed, each of the safer conception strategies provided following formative research have been feasible to implement with relatively good uptake and continuation across methods, suggesting that selected methods present important elements to be included in models proposed for scale-up. The lower uptake of PrEP indicates that greater understanding is needed on the value and potential contribution of PrEP to safer conception in a sub-Saharan African setting. While nearly half of all male partners remain uninvolved in safer conception care, reducing the potential effectiveness of safer conception services, women attending on their own may nevertheless benefit greatly through ART initiation/viral load monitoring, access to PrEP and through education around identification of the monthly time frame of peak fertility to limit condomless sex acts. Future research with safer conception clients should explore in greater depth reasons behind lower uptake of PrEP and uptake of non-recommended strategies, as well as facilitators to male partner involvement.

As universal test and treat strategies are being rolled out across sub-Saharan Africa, provision of safer conception counselling and services is now more feasible in other HIV high-burden countries in the region. In general, these strategies are low cost and can be offered without PrEP where it is not yet available. Our results suggest that efforts to include viral load monitoring, when possible, may be important to ensure HIV transmission risks are minimized.

Given the positive experiences in this demonstration project, we recommend further implementation evaluation of the effectiveness of integration of safer conception strategies into existing service delivery mechanisms as this can ensure that service delivery remains cost-efficient and meets client demand across primary health centres.
